# Long-term kidney and patient outcomes in pure versus mixed lupus membranous nephritis

**DOI:** 10.1093/ckj/sfag264

**Published:** 2026-08-17

**Authors:** Martina Uzzo, Marisol Bracalenti, Marta Calatroni, Barbara Trezzi, Emanuele Conte, Laura Locatelli, Mariele Gatto, Roberto Depascale, Giovanni Maria Rossi, Vincenzo Limperio, Renato Alberto Sinico, Andrea Doria, Gabriella Luisa Moroni

**Affiliations:** Nephrology and Dialysis Division, IRCCS Humanitas Research Hospital, Rozzano, Milan, Italy; Department of Biomedical Sciences, Humanitas University, Pieve Emanuele, Milan, Italy; Rheumatology Unit, Department of Medicine, University of Padova, Padova, Italy; Nephrology and Dialysis Division, IRCCS Humanitas Research Hospital, Rozzano, Milan, Italy; Department of Biomedical Sciences, Humanitas University, Pieve Emanuele, Milan, Italy; Nephrology and Dialysis Division, IRCCS Humanitas Research Hospital, Rozzano, Milan, Italy; Nephrology and Dialysis Division, IRCCS Humanitas Research Hospital, Rozzano, Milan, Italy; Nephrology and Dialysis Division, IRCCS Humanitas Research Hospital, Rozzano, Milan, Italy; Academic Rheumatology Centre, Department of Clinical and Biological Sciences, University of Turin, AO Mauriziano, Turin, Italy; Rheumatology Unit, Department of Medicine, University of Padova, Padova, Italy; Nephrology Unit, Department of Medicine and Surgery, University of Parma, Parma, Italy; Department of Medicine and Surgery, Pathology, Fondazione IRCCS San Gerardo dei Tintori, University of Milano-Bicocca, Monza, Italy; Nephrology and Dialysis Division, IRCCS Humanitas Research Hospital, Rozzano, Milan, Italy; Rheumatology Unit, Department of Medicine, University of Padova, Padova, Italy; Nephrology and Dialysis Division, IRCCS Humanitas Research Hospital, Rozzano, Milan, Italy; Department of Biomedical Sciences, Humanitas University, Pieve Emanuele, Milan, Italy

**Keywords:** classification, histology, kidney biopsy, lupus nephritis

## Abstract

**Objective:**

Pure membranous lupus nephritis [pMLN, Class V International Society of Nephrology/Renal Pathology Society (ISN/RPS)] is traditionally considered less aggressive than mixed forms of MLN (mMLN, Classes III/IV ± V ISN/RPS). Only two trials have compared the outcome of these two forms of MLN, with inconclusive results. In this retrospective multicentre study, we assessed and compared the short and long-term outcomes of pMLN versus mMLN.

**Methods:**

Patients diagnosed with pMLN or mMLN on their first kidney biopsy were included. The main outcomes were complete renal response after 1 year, sustained complete response, relapse rates, chronic kidney disease (CKD)-free survival, and the composite outcome of kidney failure (KF)/death-free survival.

**Results:**

Of the 195 patients included, 96 (49.2%) pMLN had milder immunological features of active LN at baseline compared to 99 (50.8%) with mMLN. At kidney biopsy, the National Institute of Health activity and chronicity indices were lower in patients with pMLN (*P *< .001). Moreover, they received less intensive immunosuppressive treatment (less i.v.-steroids, lower use/earlier withdrawal of the immunosuppressive regimen). Both groups achieved similar rates of responses, relapses, and kidney survival over follow-up.

KF/death-free survival was significantly lower in pMLN compared to mMLN (*P *= .026). In a Cox-model adjusted for baseline kidney function and induction immunosuppressive regimen, mMLN retained an 80% lower risk of KF/death compared to pMLN (*P *= .0384). In a subgroup analysis, crude survival differences between pMLN and mMLN were confirmed only in patients biopsied before 2000.

**Conclusion:**

This study challenges the long-standing view of pMLN as a generally benign condition, suggesting that less intensive treatment of pure MLN may have contributed to less favourable hard outcomes in the long-term.

## Graphical Abstract

**Graphical Abstract:**
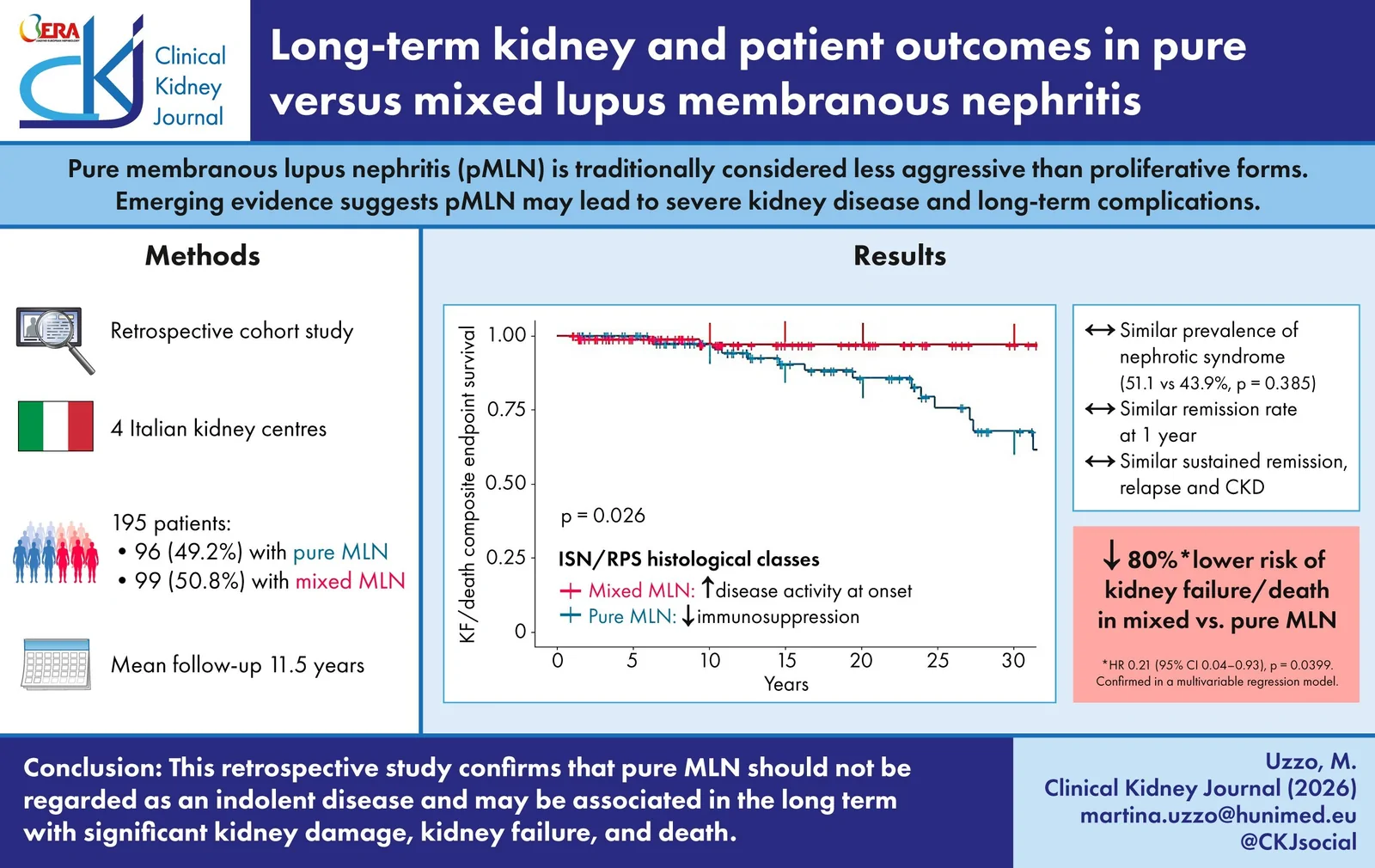

KEY LEARNING POINTS **What was known:**

- MLN is traditionally considered less aggressive than proliferative forms and presents with a lower clinico/immunological disease activity.

**This study adds:**

- Pure MLN showed a higher risk of kidney failure/death compared to mixed MLN, particularly in the earlier treatment eras, suggesting that it should not be regarded as an indolent disease.

**Potential impact:**

- The less aggressive approaches used to treat pure MLN may partially explain the higher risk of kidney failure/death compared to mixed MLN.

## INTRODUCTION

Pure membranous lupus nephritis [pMLN, Class V according to the International Society of Nephrology/Renal Pathology Society (ISN/RPS) classification [1, 2]] is a distinct subtype of lupus nephritis (LN) accounting for 15%–20% of the histological forms, characterized by unique clinical and histopathological features [3]. Traditionally considered as less aggressive than proliferative LN (Classes III and IV ISN/RPS^1^) [3, 4], pMLN commonly presents with nephrotic syndrome and carries a significant risk for thrombotic events [5, 6]. Compared to proliferative classes, it typically shows lower systemic lupus erythematosus (SLE) disease activity, in clinical, laboratory, and histological findings [5, 6].

The recommended treatment approach of pMLN varies across different guidelines, particularly for patients with sub-nephrotic proteinuria [7–9]. While emerging evidence suggests that pMLN may lead to severe kidney damage and long-term complications [10–14], randomized controlled trials exploring new treatment approaches mainly focus on proliferative classes [15–18] and only a few retrospective studies have specifically compared pMLN to mMLN, yielding conflicting results [3, 5].

This retrospective, multicentre study aimed to analyse and compare baseline characteristics, as well as short- and long-term clinical outcomes and response to therapy, between patients with pMLN and mMLN.

## MATERIALS AND METHODS

### Study population

Patients diagnosed with SLE according to the 2019 American College of Rheumatology (ACR) criteria were included [19]. Eligible patients had a histopathological diagnosis of pure or mixed MLN—defined as Class V and Classes V + III or V + IV 1 ISN/RPS, respectively—based on their first kidney biopsy performed between 1968 and 2023. All patients had a minimum follow-up of 1 year after the biopsy. Patients on chronic renal replacement therapy or who underwent previous kidney transplantation were excluded. Data were collected from four referral SLE Italian centres: the Nephrology Unit of IRCCS Humanitas Research Hospital (Rozzano), the Nephrology Unit of IRCCS San Gerardo Hospital (Monza), the Rheumatology Unit of Padua University-Hospital (Padua), and the Nephrology Unit of Parma University Hospital (Parma).

### Ethical approval

The study was performed in accordance with the declaration of Helsinki and was approved by the Ethics Committee of the IRCCS Humanitas Research Hospital, Milan, Italy (protocol number NEF0032023). All patients provided informed consent for the scientific use of their anonymized data. Patient or public involvement in the research was not applicable.

### Data collection

The patients’ clinical and demographic variables were prospectively recorded in a dedicated database. The following variables were collected: (1) clinical/demographic features and laboratory tests at the time of histopathological LN diagnosis; (2) kidney biopsy reports; (3) follow-up data at 1 year and at the latest clinical evaluation. Kidney biopsy reports were collected retrospectively and classified according to the ISN/RPS 1 classification; the National Institutes of Health (NIH) activity and chronicity indices [1] were recorded. Estimated glomerular filtration rate (eGFR) was calculated using the Chronic Kidney Disease Epidemiology Collaboration formula. Induction therapies, whether based on glucocorticoids (GCs), immunosuppressive regimens, or both, were recorded.

### Outcome measures and definitions

Included patients were divided into two groups according to the histopathological classification at their first kidney biopsy: (1) patients with pMLN (Class V ISN/RPS^1^); (2) patients with mMLN (Classes V + III or V + IV ISN/RPS^1^). Outcomes were evaluated in the overall cohort and according to the histopathological classification (pMLN vs. mMLN). The main outcomes were (a) complete renal response (CRR) at 1 year after kidney biopsy, (b) sustained complete response (SCR), (c) renal relapse rate (d) chronic kidney disease (CKD) free survival and the composite outcomes of CKD/death and kidney failure (KF)/death free survival. A subgroup analysis was performed on two historical groups, divided according to kidney biopsy timing (between 1968 and 1999 and between 2000 and 2023). The two periods aim at reflecting changes in LN management occurring from the early 2000s, due to the introduction of mycophenolate mofetil and new therapeutic strategies [20].

The following definitions were used: CKD, eGFR <60 ml/min/1.73 m2 for at least 3 months; KF, GFR <15 ml/min per 1.73 m^2^ or need for chronic renal replacement therapy until the last available follow-up (at least 3 months); nephrotic syndrome, proteinuria >3.5 g/day with plasma albumin <3 g/dl; non-nephrotic proteinuria, between 0.5 and 3.5 g/day [21]; urinary abnormalities, abnormal proteinuria (>0.5 g/day) and/or urine sediment positive for acanthocytes (≥5%), red blood cell casts or white blood cell casts [8]; CRR, normal or near-normal (within 10% of normal eGFR if previously abnormal) kidney function and proteinuria <0.5 g/day, no active extrarenal manifestations; partial renal response (PRR), normal or near-normal (within 10% of normal eGFR if previously abnormal) kidney function and <50% decrease of baseline proteinuria, or proteinuria <3.5 g per day if nephrotic, no active extrarenal manifestations [22]; SCR, the achievement of CRR for at least 1 year [23].

Disease flares occurred in patients who had previously reached CRR and included: nephritic syndrome/flare—an increase in glomerular haematuria by ≥10 red blood cells/hpf (high power field, 400×) with a decrease in eGFR by ≥10%, irrespective of changes in proteinuria; proteinuric flare, an increase in proteinuria without modification of kidney function (at least 2 g/day if the basal proteinuria was less than 3.5 g/day, or double if the patient already had nephrotic proteinuria) or extrarenal flare [23, 24].

### Data analysis

All the analyses were performed with R 4.4.2 statistical package (https://www.R-project.org/). *P* value < .05 was regarded as statistically significant. Baseline pairwise differences in the pure versus mixed MLN groups were tested through the Mann-Whitney test for continuous variables and the Fisher’s exact for categorical variables. Crude patients’ rate of SCR, crude CKD, relapse free survival and the CKD/death and KF/death composite outcomes between groups were compared using the Kaplan-Meier estimator and log-rank test. The same method was used to estimate the crude patients’ rate of KF/death in the two historical periods, divided according to the timing of kidney biopsy performance (1968–99 and 2000–23).

A multivariable effect-size Cox regression model was employed in the overall cohort to assess the relationship between the histopathological groups and pre-defined potential confounders in predicting the KF/death composite outcome. A step AIC selection model was used to identify the best multivariable model. The linearity of continuous variables was assessed using martingale residuals, and variables were log-transformed when necessary. Schoenfeld residuals were used to test proportional Hazard assumption. Due to the low number of events for the KF/Death composite outcome (*n* = 18), interaction analyses were not performed.

## RESULTS

### Characteristics of the study population

**Figure 1:**
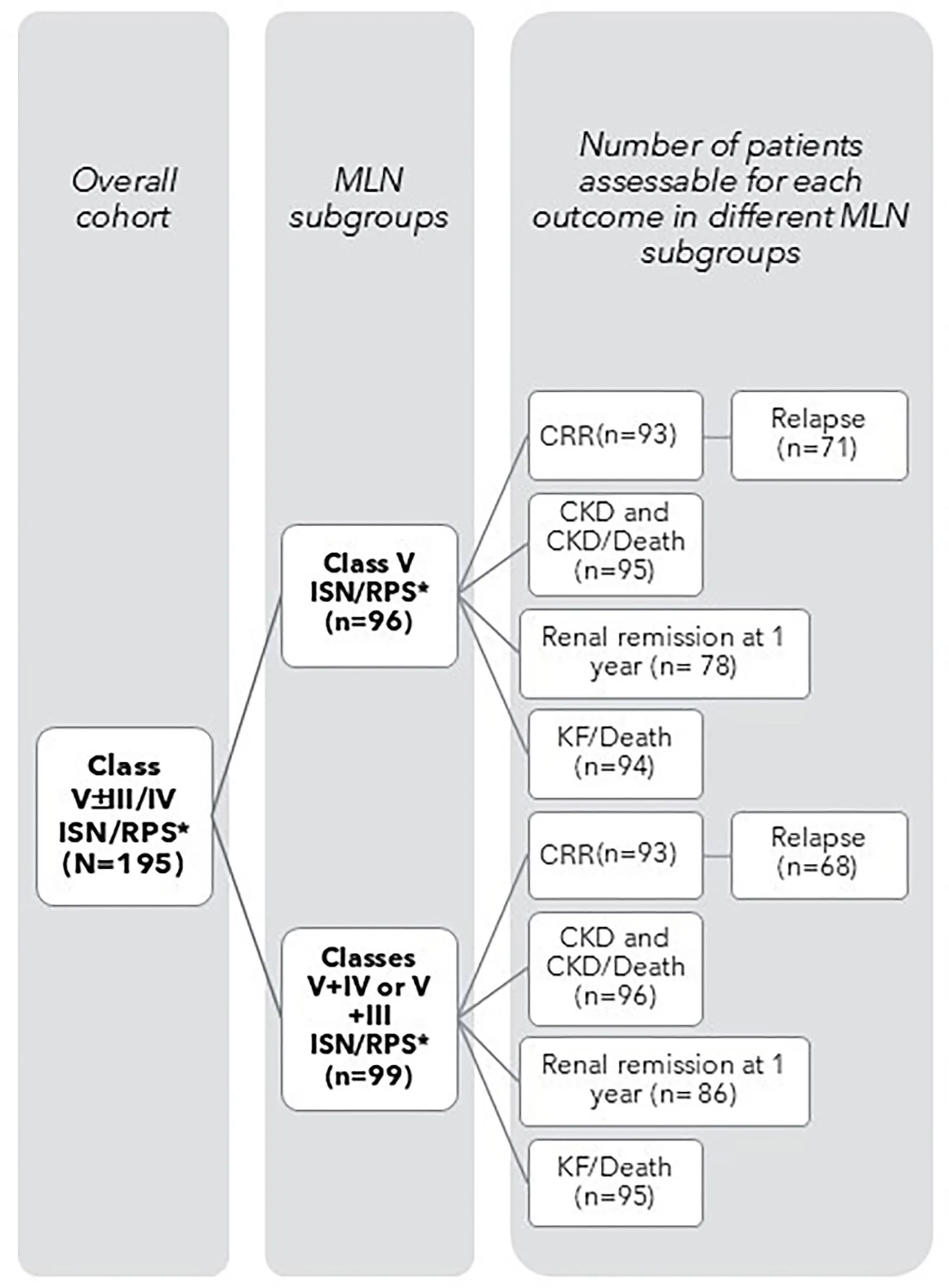
Number of patients assessable for each outcome. A cohort of 195 patients with pure or mixed MLN was eligible for the study (see text for further details). Patients were divided into two subgroups, according to the histopathological classification: 96 (49.2%) patients were classified as pure MLN (Class V ISN/RPS1); 99 (50.8%) patients were classified as mixed MLN (Classes V + III and V + IV ISN/RPS1). MLN, membranous lupus nephritis; CRR, complete renal response; CKD, chronic kidney disease; KF, kidney failure.

The 195 eligible patients were divided into two groups according to the ISN/RPS1 histopathological classification: 96 (49.2%) were classified as pure MLN (Class V ISN/RPS^1^), and 99 (50.8%) as mixed MLN (Classes V + III and V + IV ISN/RPS^1^). Figure 1 shows the number of patients computable for each outcome, while Table 1 reports baseline features of the overall cohort and of the two histopathological groups. The overall median follow-up time was 11.50 (IQR 6.19, 21.92) years, with a significant difference between pure and mixed MLN groups [13.97 (IQR 7.01, 23.97) years vs. 9.48 (IQR 4.83, 18.98) years, respectively; *P *= .005]. The median age of patients enrolled in the cohort was 31.44 (IQR 23.63, 41.14) years, with female predominance (*n* = 163, 83.6%). More common extrarenal symptoms in the overall cohort included joint (*n* = 135, 70.3%) and skin (*n* = 106, 54.9%) involvement. Out of 195 patients, 91 (47.4%) had nephrotic syndrome as a presenting feature, 15 (7.8%) had nephritic syndrome, and 86 (44.8%) had isolated urinary abnormalities. Nephritic syndrome was more frequent in patients with mMLN compared to pMLN (14.3 vs. 1.1%, *P *< .001). Accordingly, the mMLN group exhibited a higher number of red blood cells per high-power field (hpf, 400×) in the urinary sediment (15 vs. 5 cells/hpf, *P *= .001). The median eGFR at baseline in the overall cohort was 106.5 (IQR 89.25, 121.00) ml/min/1.73 m2, and the median urine protein level was 3.60 (IQR 2.06, 5.48) g/day, without differences between histological groups. At the kidney biopsy, mMLN had higher NIH activity and chronicity indices [1] compared to pMLN [activity index: 6 (IQR 4, 8) vs. 0 [0, 2), *P *< .001; chronicity index: 2 (IQR 1, 4) vs. 1 (IQR 0, 2), *P *< .001].

**Table 1:** Baseline characteristics of a cohort of MLN patients at the time of kidney biopsy.

	Pure MLN (*n* = 96)	Mixed MLN (*n* = 99)	Total (*N* = 195)	*P* value
**(a) Demographic, kidney features**				
Race (*n*, %)				.336
Caucasian	85 (88.5%)	78 (78.8%)	163 (83.6%)	
American Indian	4 (4.2%)	9 (9.1%)	13 (6.7%)	
Asian	4 (4.2%)	6 (6.1%)	10 (5.1%)	
African	3 (3.1%)	6 (6.1%)	9 (4.6%)	
Sex (*n*, %)				.848
Female	81 (84.4%)	82 (82.8%)	163 (83.6%)	
Time from SLE to kidney diagnosis (yr)				**.044**
Median (Q1, Q3)	0.54 (0.00, 4.99)	1.37 (0.08, 6.94)	1.00 (0.00, 5.45)	
Age at kidney biopsy (yr)				.700
Median (Q1, Q3)	31.36 (23.53, 41.02)	31.83 (23.82, 41.14)	31.44 (23.63, 41.14)	
Activity index ISN/RPS*				**<.001**
Median (Q1, Q3)	0 (0, 2)	6 (4, 8)	3 (0, 6)	
Chronicity index ISN/RPS*				**<.001**
Median (Q1, Q3)	1 (0, 2)	2 (1, 4)	1 (0, 3)	
Kidney disease (*n*, %)				**.003**
Urinary abnormalities	45 (47.9%)	41 (41.8%)	86 (44.8%)	.386
Nephrotic syndrome	48 (51.1%)	43 (43.9%)	91 (47.4%)	.385
Nephritic syndrome	1 (1.1%)	14 (14.3%)	15 (7.8%)	**<.001**
eGFR (CDK-EPI) (ml/min/1.73 m^2^)				.116
Median (Q1, Q3)	110.00 (90.00, 125.00)	104.00 (88.00, 119.00)	106.50 (89.25, 121.00)	
Proteinuria (g/day)				.085
Median (Q1, Q3)	3.08 (1.86, 5.30)	3.90 (2.40, 5.60)	3.60 (2.06, 5.48)	
Red cells urine (400×)				**.001**
Median (Q1, Q3)	5.00 (1.00, 14.00)	15.00 (4.25, 30.00)	10.00 (2.00, 21.50)	
Hypertension (*n*, %)	30 (31.2%)	39 (39.4%)	69 (35.4%)	.442
Albumin (g/dl)				.359
Median (Q1, Q3)	2.90 (2.39, 3.40)	2.93 (2.52, 3.50)	2.90 (2.40, 3.50)	
C3 (g/l)				**.039**
Median (Q1, Q3)	73.25 (56.62, 92.50)	66.50 (49.25, 79.00)	69.00 (51.75, 90.00)	
C4 (g/L)				**.002**
Median (Q1, Q3)	13.05 (9.25, 21.75)	10.00 (6.00, 15.00)	12.00 (7.00, 19.10)	
**(b) Extrarenal involvement, initial therapy**				
No treatment (no steroids, no IS regimen)	6 (6.3%)	0 (0%)	6 (3.1%)	**.03**
Steroids without IS regimen	16 (16.7%)	8 (8.1%)	24 (12.3%)	.108
IS regimen without steroids	3 (3.1%)	4 (4.0%)	7 (3.6%)	1
Steroids plus IS regimen	71 (73.9%)	87 (87.9%)	158 (81.0%)	**.02**
Steroids (*n*, %)				**<.001**
(no steroids)	9 (9.4%)	4 (4.0%)	13 (6.7%)	.159
Steroids ev + oral	63 (65.6%)	82 (82.8%)	145 (74.4%)	**.008**
Steroids oral only	14 (14.6%)	1 (1.0%)	15 (7.7%)	**<.001**
Steroids oral (ev not known)	10 (10.4%)	12 (12.1%)	22 (11.3%)	.822
Immunosuppressive regimen—induction (*n*, %)				**<.001**
(no immunosuppressive regimen)	22 (22.9%)	8 (8.1%)	30 (15.4%)	**.002**
CYC	38 (39.6%)	41 (41.8%)	79 (40.6%)	.884
AZA	6 (6.2%)	3 (3.1%)	9 (4.6%)	.326
MMF	20 (20.8%)	38 (38.8%)	58 (29.7%)	**.008**
CyA	4 (4.2%)	3 (3.1%)	7 (3.6%)	.718
Others	4 (6.3%)	5 (5.1%)	9 (6.2%)	1.000
Hydroxychloroquine (*n*, %) (introduced at the time of induction)	26 (29.2%)	46 (48.9%)	72 (39.3%)	**.007**
Autoimmunity (*n*, %)				
Anti-dsDNA	64 (70.0%)	84 (86.5%)	148 (78.7%)	**.011**
Antiphospholipid	28 (33.7%)	25 (27.4%)	53 (34.4%)	.464
anti-Smith	18 (23.1%)	25 (28.4%)	43 (25.9%)	.481
anti-SSA	26 (34.7%)	27 (30.3%)	53 (32.3%)	.616
anti-SSB	8 (10.8%)	10 (11.2%)	18 (11.0%)	1.000
anti-Rnp	20 (26.0%)	28 (31.5%)	48 (28.9%)	.494
Organ involvement (*n*, %)				
Lymph nodes	10 (10.8%)	13 (13.4%)	23 (12.1%)	.659
Skin	53 (56.4%)	53 (53.5%)	106 (54.9%)	.773
Joints	58 (61.7%)	77 (78.6%)	135 (70.3%)	**.012**
Serous membranes	26 (27.7%)	22 (22.2%)	48 (24.9%)	.409
Central Nervous System	5 (5.3%)	6 (6.2%)	11 (5.8%)	1.000
Fever	43 (45.7%)	39 (40.2%)	82 (42.9%)	.467

Baseline characteristics refer to the time of kidney biopsy. Continuous variables are reported as median (IQR), categorical variables as number (%).

eGFR, estimated glomerular filtration rate; CDK-EPI, Chronic Kidney Disease Epidemiology Collaboration formula; ISN, International Society of Nephrology; RPS, Renal Pathology Society; MLN, membranous lupus nephritis; Anti-dsDNA, autoantibodies anti-double strand DNA; LAC, lupus anticoagulant; MMF, mycophenolate mofetil; CYC, cyclophosphamide; AZA, azathioprine; CyA, cyclosporine; IS, immunosuppressive regimen.

Data from the overall cohort, the pure (ISN/RPS1 class V) and the mixed (ISN/RPS1 classes V + III and V + IV) subgroups.

*ISN/RPS [1].

Moreover, mMLN had lower levels of complement [C3 66.50 (IQR 49.25, 79.00) g/l vs. 73.25 (IQR 56.62, 92.50) g/l, *P *= .039; C4 10.00 (IQR 6.00, 15.00) g/l vs. 13.05 (IQR 9.25, 21.75) g/L, *P *= .002], a higher prevalence of positive anti-dsDNA antibodies (86.5 vs. 70.0%, *P *= .011) and a higher prevalence of joint involvement compared to pMLN (78.6 vs. 61.7%, *P *= .012) **(**Table 1a).

Overall, 182 (93.3%) patients received GC at the time of initial therapy; among these, 158 (81.0%) received both steroids and immunosuppressive regimen. A significantly lower proportion of patients with pMLN was treated with intravenous methylprednisolone pulses followed by oral GC compared to mMLN (65.6 vs. 82.8%, *P *= .008). Moreover, a lower proportion of pMLN patients received both GC and immunosuppressive regimen as initial therapy (73.9.1 vs. 87.9%, *P *= .02). In the pMLN group, 6 patients (6.3%) were managed with supportive therapy, without GC and immunosuppressants, while all mMLN patients received GC and/or immunosuppressants. Among the various immunosuppressive agents, mycophenolate mofetil (MMF) was more commonly administered in mMLN than in pMLN (38.8 vs. 20.8%, *P *= .008) and hydroxychloroquine was taken by 48.9% of patients with mMLN, compared to 29.2% of those with pMLN (*P *= .007) (Table 1b).

### Complete response at 1 year, sustained complete response and risk of relapse

One year after kidney biopsy, 81 (49.4%) patients achieved CRR and 58 (35.3%) achieved PRR. No differences in terms of renal response at one year or in terms of clinical features were detected between pMLN and mMLN [CRR 37 (47.4%) vs. 44 (51.2%) and PRR 28 (35.9%) vs. 30 (34.9%), respectively; *P *> .05].

Importantly, a higher proportion of pMLN patients withdrew the immunosuppressive regimen within the first year (19.0 vs. 4.4%, *P *= .008) **(**Supplementary Table S1). Baseline clinical and laboratory data of patients who had withdrawn the immunosuppressive regimen within 1 year (*n* = 20) and those which have never started the immunosuppressive regimen (*n* = 30) are reported in Supplementary Table S2: of note, no patients in the mMLN group had suspended steroids at one year, while 11 (31.4%) patients in the pMLN group did.

In the overall cohort, SCR was achieved by 143 patients (73.3%) within a median of 1.50 (IQR 0.90–3.56) years, with no significant differences between pMLN and mMLN in terms of prevalence (SCR 73.3 vs. 72.7%, *P *= .684), time to SCR (1.50 vs. 1.51 years, *P *= .513), GC dose, or duration of response (Supplementary Table S3).

SCR was achieved by 143 patients (73.3%) within a median of 1.50 (IQR 0.90–3.56) years, with no significant differences between pMLN and mMLN in terms of prevalence (SCR 73.3 vs. 72.7%, *P *= .684), time to SCR (1.50 vs. 1.51 years, *P *= .513), GC dose, or duration of response (Supplementary Table S3).

**Figure 2:**
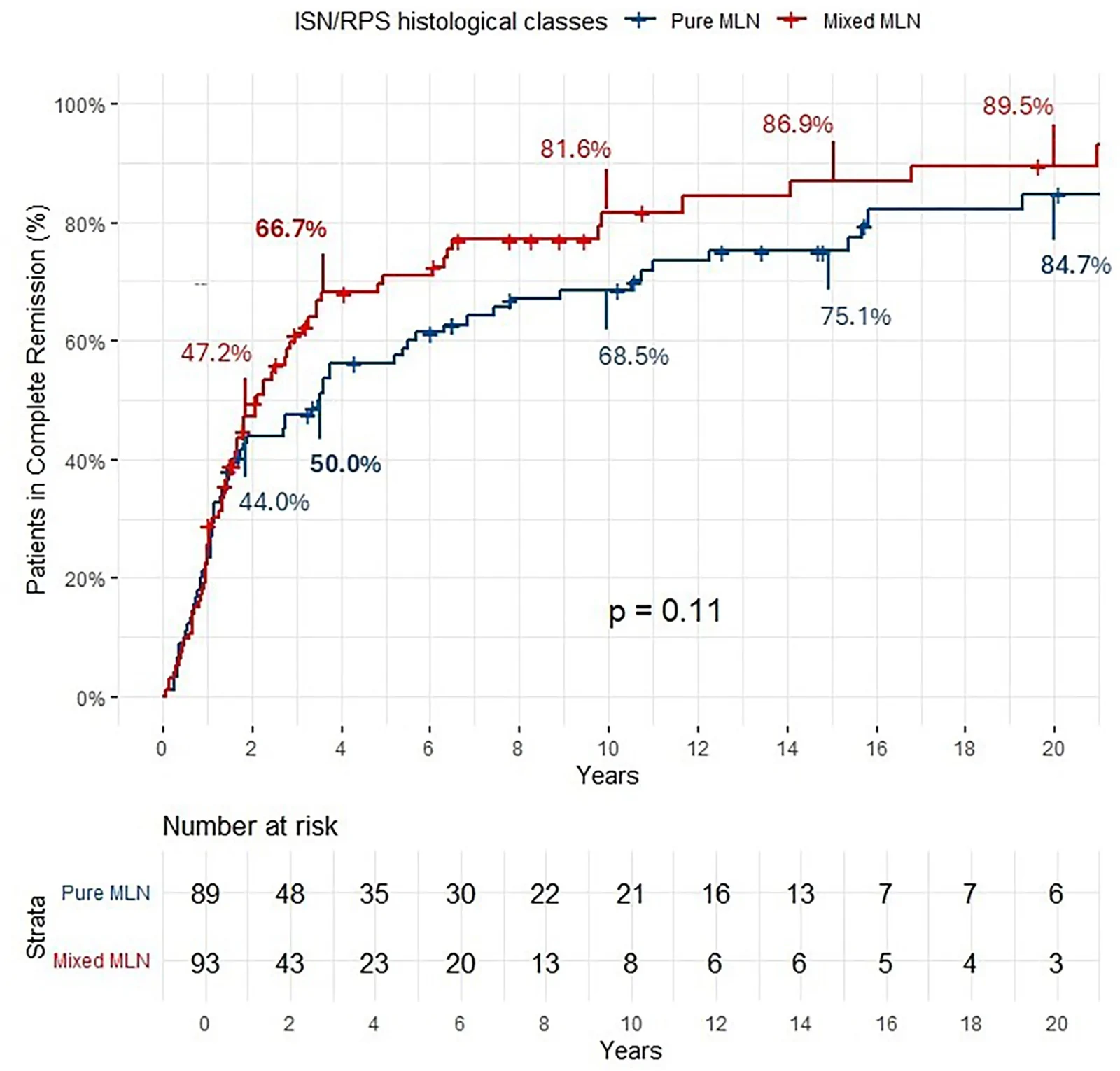
Crude rate of complete response (Kaplan-Meier estimator) in patients with pure MLN (blue line) and in those with mixed MLN (red line). *P* value refers to Log-rank test, which is based on the time to the latest available follow-up, though the plot refers to the first 20 years. MLN, membranous lupus nephritis.

Figure 2 shows that the crude rate of SCR over time was similar between the two groups (*P *= .11). Despite lacking sequential data for a formal kinetic analysis, the Kaplain Maier curve draw a trend towards a more rapid achievement of SCR in mMLN compared to pMLN patients. The peak difference was observed after 3.5 years, when 66.7% mMLN versus 50.0% pMLN patients were in SCR.

Among patients who had previously achieved SCR, 35 experienced a relapse in the overall cohort: 49.3% in the pure and 51.5% in the mixed MLN group (*P *= .865) (Supplementary Table S3). Out of these, 12 (19.7%) patients achieved SCR for the second time. Crude relapse-free survival did not differ between groups either (*P *= .45) (Supplementary Fig. S1a), even when focusing on proteinuric relapse (*P *= .26) (Supplementary Fig. S1b).

### Chronic kidney disease, kidney failure, and death from all causes

18 (18.9%) patients developed CKD during follow-up in the pMLN group, compared to 9 (9.4%) patients in the mMLN one (*P *= .064). Moreover, 6 (6.2%) patients in the first and 1 (1.0%) in the latter group developed KF (*P *= .061). Ten (10.4%) patients died in the pMLN group while two (2.0%) patients died in the mMLN one (*P *= .017) and the median time to death was 20.56 (IQR 10.12–29.33) years. Cause of death was available for 11 patients: cancer [*n* = 5 (45%)] and cerebrovascular events [*n* = 4 (36%)] were most common, with no differences between histopathological subgroups. Crude patient’s CKD-free survival did not differ significantly between the two groups (*P *= .23) (Supplementary Fig. S2a).

**Figure 3:**
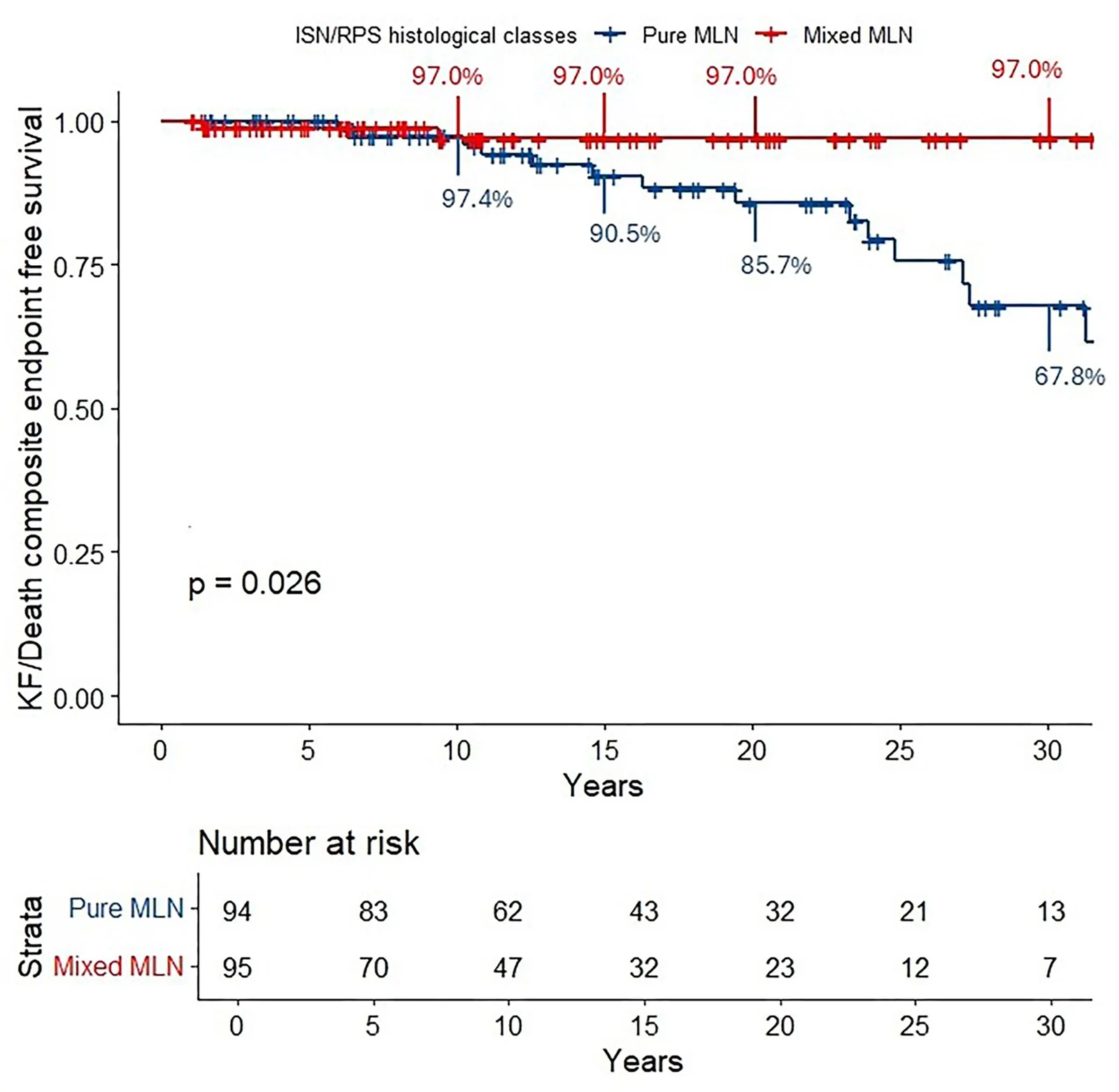
Crude patient’s Kidney Failure/death free-survival (Kaplan-Meier estimator) in patients with pure MLN (blue line) and in those with mixed MLN (red line). *P* value refers to Log-rank test, which is based on the time to the latest available follow-up, though the plot refers to the first 30 years. MLN, membranous lupus nephritis; KF, kidney failure.

To further explore long-term kidney and patients’ survival, both crude and adjusted survival rates were analysed for two composite hard endpoints: CKD combined with death (CKD/death) and KF combined with death (KF/death). Supplementary Fig. S2b shows that the crude rate of CKD/death free survival over time was numerically, but not statistically lower in the pure compared to mixed MLN group (*P *= .08). Figure 3 shows that the crude rate of KF/death free survival was lower in the pure compared to mixed MLN group (*P *= .026), with the curves diverging after 10 years of follow-up.

### Explaining ISN/RPS class effect on kidney failure/death

Pre-defined potential confounders were included in a multivariable Cox-regression model together with the histological class: baseline eGFR (ml/min/1.73 m^2^, log transformed for linearity), baseline proteinuria (g/day), the use of immunosuppressive regimen beyond GC at the time of initial therapy, and kidney biopsy date (before or after 2000).

The best effect-size multivariable model explaining the impact of histopathological class on KF/death, included baseline eGFR and the use of immunosuppressants as initial therapy, both of which emerged as significant predictors of the outcome [HR 0.22 (CI 95% 0.06–0.79), *P *= .0210 and HR 0.32 (CI 95% 0.11–0.90), *P *= .0318, respectively]. Within this adjusted model, the mMLN group remained associated with an 80% lower risk of KF/death compared to the pMLN group [HR 0.20 (CI 95% 0.04–0.91), *P *= .0384]. The ISN/RPS histological class thus persisted in this cohort as an independent predictor of KF/death, with its effect largely unchanged after adjustment. Univariable and multivariable analyses are reported in Table 2.

**Table 2:** Crude and adjusted KF/death free-survival in a cohort of MLN patients.

*Unadjusted analysis*	HR (CI 95%)	*P*
Mixed MLN classes (ISN/RPS^a,c^)	**0.21 (0.04–0.93)**	.**0399**
Immunosuppressive regimen (induction therapy)^b^	0.39 (0.14–1.07)	.0701
eGFR (CKD-EPI) (ml/min/1.73 m^2^)	0.35 (0.10–1.18)	.0935
Proteinuria (g/day)	0.99 (0.97–1.01)	.5980
Kidney biopsy timing (before 2000)	0.23 (0.04–1.14)	.0730
*Adjusted analysis*	HR	*P*
Mixed MLN classes (ISN/RPS^a,c^)	**0.20 (0.04–0.91)**	.**0384**
Immunosuppressive regimen (induction therapy)^b^	**0.32 (0.11–0.90)**	.**0318**
eGFR (CKD-EPI) (ml/min/1.73 m^2^)	**0.22 (0.06–0.79)**	.**0210**

A multivariable effect-size Cox regression model was employed in the overall cohort to assess the relationship between the histopathological groups (pure vs. mixed MLN, per ISN/RPS [1]), and pre-defined potential confounders in predicting the KF/Death composite outcome. A step AIC selection model was used to identify the best multivariable model for the prediction of outcome. In the best model, baseline eGFR (ml/min/1.73 m^2^, log transformed for linearity) and the use of immunosuppressive regimen for induction were selected as covariates.

Within this adjusted model, the mixed MLN group remained associated with an 80% lower risk of KF/Death compared to the pure MLN group (HR 0.20 (CI 95% 0.04–0.91), *P *= .0384). These findings confirm the ISN/RPS histological class as an independent predictor of KF/Death, with its effect remaining largely unchanged in the adjusted model. Cox regression model adjusted for potential confounders. Data are HRs for Cox regression model.

^a^HRs refer to mixed versus pure MLN classes.

^b^HRs refer to the use or not of the immunosuppressive regimen as induction therapy. HRs refer to the kidney biopsy timing before 2000 versus after 2000.

CKD, chronic kidney disease; KF, kidney failure; ISN, International Society of Nephrology; RPS, Renal Pathology Society, MLN, Membranous Lupus Nephritis.

^c^ISN/RPS [1].

To further explore the impact of kidney biopsy timing—based on the hypothesis that the effect of the historical period might not be constant over time and therefore may not be adequately captured by the Cox proportional hazards model—the crude survival rates for KF/death were analysed in two subgroups defined by biopsy timing. A total of 61 patients (31.3%) underwent biopsy between 1968 and 1999, while 134 patients (68.7%) were biopsied between 2000 and 2023.

**Figure 4:**
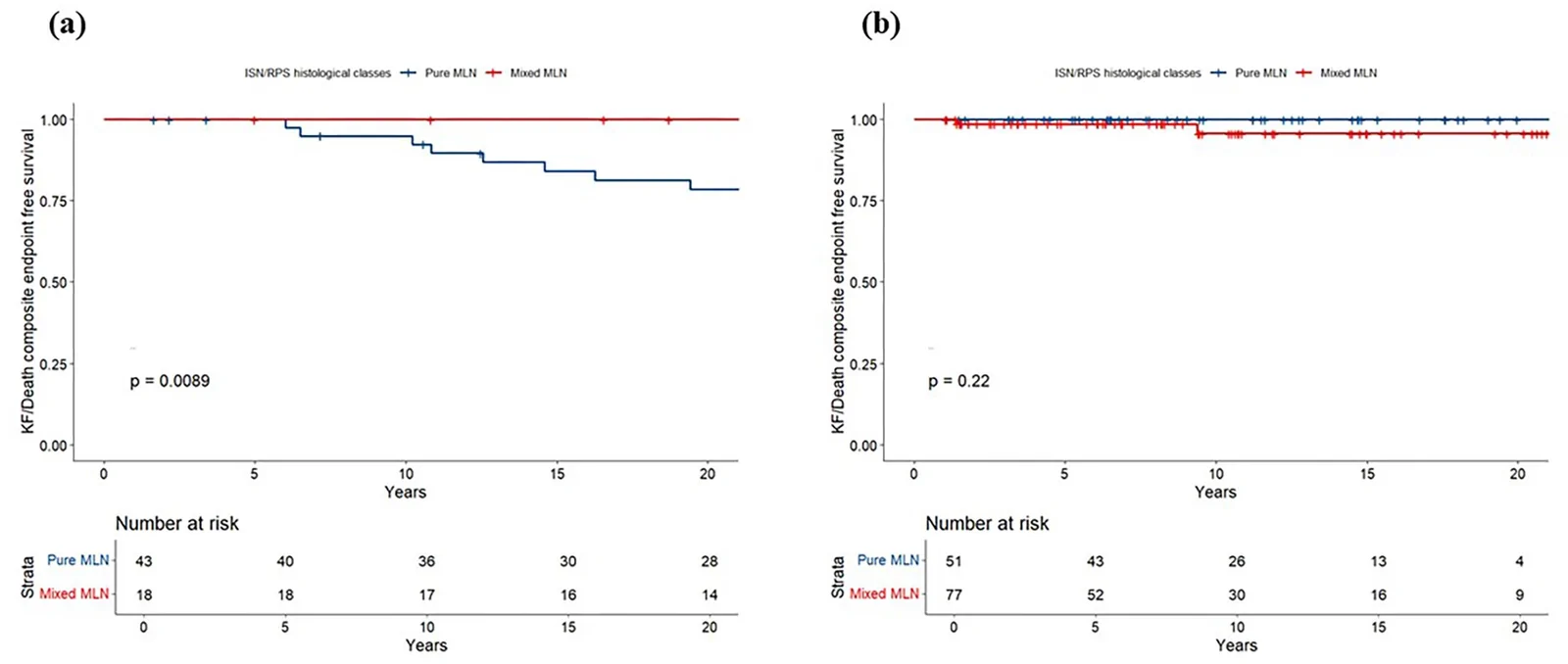
Crude patient’s Kidney Failure/death free-survival (Kaplan-Meier estimator) in patients with pure MLN (blue line) and in those with mixed MLN (red line), according to the kidney biopsy timing. (a) Kidney biopsy performed between 1968 and 1999; (b) Kidney biopsy performed between 2000 and 2023. *P* value refers to Log-rank test, which is based on the time to the latest available follow-up, though the plot refers to the first 30 years. MLN, membranous lupus nephritis; KF, kidney failure.

Figure 4 shows that the crude rate of KF/death-free survival differed significantly between the two histopathological groups when the kidney biopsy was performed between 1968 and 1999 (*P *= .0089), with pMLN confirming a worse prognosis. In contrast, no significant differences were observed between groups when the biopsy was performed between 2000 and 2023 (*P *= .22). The median follow-up time of patients diagnosed between 1968 and 1999 was similar in the mixed and pure MLN group [24.85 (IQR 12.51, 31.27) vs. 26.85 (IQR 24.23, 31.39) years, respectively; *P *= .402]. In this second period, despite a comparable proportion of patients receiving an immunosuppressive regimen at induction, only 26 (60.5%) patients received both intravenous and oral GC in the pMLN group, compared to 16 (88.9%) patients in the mMLN. Baseline characteristics of patients with pure or mixed MLN in different historical periods are reported in Supplementary Table S4 and S5.

## DISCUSSION

This is the largest study to date comparing the clinical features and the short- and long-term outcomes of pure versus mixed MLN, with an extensive follow-up period spanning over five decades. Despite presenting with fewer clinical and immunological features of active LN at baseline, patients with pure MLN achieved similar rates of complete renal response at one year, similar SCR over time, and similar relapse rates to those with mixed MLN.

Notably, the proportion of patients presenting with nephrotic syndrome was similar in both groups (*P *= .385). However, by the end of the observation period, the pure MLN group showed a lower KF—and death-free survival compared to the mixed MLN one, questioning the long-standing view of a generally benign condition.

The current opinion has long held that pure MLN carries a better prognosis than mixed MLN [3, 5]. However, only two studies have specifically compared pure versus mixed MLN. The first collaborative study, conducted in Chicago in 1996 by Sloan *et al*., reported a better 10-year kidney survival in the pure MLN group compared to the mixed MLN group (72 vs. 20%, *P *< .05) [3]. Nonetheless, this study was limited by a short mean follow-up (5.8 vs. 2.7 years in the two groups, respectively), a small sample size (79 patients), and reliance on historical data [3]. The second study, conducted in Italy by Moroni *et al*., which laid the groundwork for the present investigation, included 103 patients and found no significant differences between the 67 patients with pure MLN and the 36 with mixed MLN, either in terms of kidney response or patient/kidney survival at 10 years (94.5 vs. 94.0% and 85.8 vs. 86.0%, respectively) [3, 5].

The present study is an updated, long-term extension study of the original cohort of 103 patients which has expanded to 195 patients. In this cohort, consistently with previous data [5, 6], mixed MLN patients presented with more active immunological disease profile at onset compared to pure MLN ones. Despite differences in baseline features, the two groups showed comparable outcomes in terms of CRR at 1 year, time to SCR, relapse-free survival, and CKD-free survival. As previously reported, the Kaplain Maier curve described a trend towards a more rapid achievement of SCR in mixed MLN compared to pure MLN patients, with the largest difference observed in our cohort after 3.5 years [5, 25] (Fig. 2). Notably, an earlier resolution of proteinuria has been associated with better long-term kidney outcomes [26, 27]. If confirmed in kinetics analyses, this phenomenon could potentially be explained by the more severe podocyte damage observed in pMLN forms, which may require a longer healing period. Alternatively, the more aggressive treatment approach used in the mixed forms may result in a faster response during the early stage of glomerular inflammation.

In this cohort, a substantial proportion of patients—particularly those with pure MLN enrolled between 1968 and 1999—were treated with oral GCs alone, reflecting the prevailing belief at the time that pure MLN had an indolent course. In this subgroup, the KF/death composite end point was significantly poorer (*P *= .0089). In contrast, survival curves were largely overlapping among patients who underwent kidney biopsy between 2000 and 2023, with no significant difference between groups (*P *= .22). The median follow-up time during this historical period was similar between the pure and mixed MLN groups. However, since the early 2000s, the introduction of mycophenolate in LN treatment has revolutionized disease management. Indeed, most pure MLN patients enrolled in this period received immunosuppressive drugs and/or methylprednisolone pulses.

Moreover, in a larger proportion of pure MLN patients -mostly enrolled between 1968 and 1999- the immunosuppressive regimen was discontinued within the first year (*P *= .008). As a result, the long-term maintenance therapy currently recommended for LN [28] was not administered. These observations support the hypothesis that advancements in management strategies have contributed to improved outcomes in MLN.

In 2009, a small randomized controlled trial first demonstrated that combining prednisone with cyclophosphamide or cyclosporine improved response rates compared to GC alone. To date, the study by Austin *et al*. remains the only randomized controlled trial specifically focused on pure MLN, providing the first high-quality evidence supporting the addition of immunosuppressive agents to GC in managing this condition [29].

The 2019 EULAR recommendations were the first to suggest the use of immunosuppressive therapy in pure MLN patients with proteinuria >1 g/day [18]. Furthermore, the recently published ACR guidelines for LN now recommend a triple therapy consisting of corticosteroids, mycophenolate, and calcineurin inhibitors, even in cases of pure MLN [9]. For the first time, the updated EULAR recommendations do not propose distinct treatment approaches for pure MLN versus proliferative LN [18]. Moreover, although patients with pure MLN were not included in the REGENCY trial—and remain consistently underrepresented in LN randomized controlled trials—the 2025 EULAR guidelines now suggest obinutuzumab as a valuable treatment option [16, 18].

Despite being the largest study to date directly comparing pure and mixed MLN, limitations warrant caution in interpreting the results. Its retrospective design—although in prospectively collected data—introduces the potential for selection and information biases. First, the therapeutic approach has been decided based on clinical judgement and has changed over the years: immunosuppressive treatment was modelled only as an induction baseline variable, without accounting for cumulative exposure, maintenance duration, later escalation, or time-varying treatment changes.

Second, our paper does not provide granular or model-based analysis of proteinuria or eGFR slopes and response definitions may compress trajectory information; moreover, the limited number of KF/death events is an important constraint and did not allow to perform interaction analyses. Unfortunately, the follow-up time was statistically different in the pure versus the mixed groups, leading to potential bias; however, the two histopathological groups had the same follow-up time when divided into historical subgroups. Most data were derived from histopathological reports, which may not always reflect current diagnostic standards, particularly for older biopsies, the lack of central evaluation prevented to use new emerging biomarkers on kidney biopsies, such as exostosin 1 and 2 [30–32].

In conclusion, this large retrospective study indicates that pure MLN should not be considered uniformly benign in the long term, as it may be associated with significant kidney damage, KF, and death. On the one hand, historically less aggressive treatment strategies for pure MLN—such as relying solely on GC, avoiding additional immunosuppressive therapy, or omitting maintenance immunosuppression after induction—may have contributed to the differences in long-term outcomes observed when compared with mixed MLN; on the other hand, the absence of significant survival differences in the modern era argues against an intrinsically more aggressive biological course. These findings support currently updated recommendations to avoid undertreatment of clinically active pure MLN and underscore the need for a more personalized approach to MLN management, integrating both clinical and pathological features to better stratify risk and guide therapeutic decisions.

## Supplementary Material

sfag264_Supplemental_Files

## Data Availability

The data that support the findings of this study are available on request from the corresponding author, MU. The data are not publicly available due to information that could compromise the privacy of research participants.
