# Supplementary material for: Long-term kidney and patient outcomes in pure versus mixed lupus membranous nephritis
Source: Clin Kidney J. 2026 Aug 17;19(9):sfag264. doi: 10.1093/ckj/sfag264 (PMC13572870; doi:10.1093/ckj/sfag264)
Supplement: Supplementary file 1 [file sfag264_supplemental_files.zip › Supplemental material.pdf]

Supplemental material

Supplementary Table 1 – Response to therapy and clinical characteristics one-year post-kidney biopsy in a cohort of MLN patients. Data from the overall cohort, the pure (ISN/RPS[1] class V) and the mixed (ISN/RPS[1] classes V+III and V+IV) subgroups.

	Pure MLN (n=96)	Mixed MLN (n=99)	Total (N=195)	p value
**Renal response at 1 year**				0.803
Complete renal response^	37 (47.4%)	44 (51.2%)	81 (49.4%)	
Partial renal response°	28 (35.9%)	30 (34.9%)	58 (35.4%)	
No renal response	13 (16.7%)	12 (14.0%)	25 (15.2%)	
**Clinical features at 1 year**				
**Proteinuria (g/day)**				0.678
Median (Q1, Q3)	0.75 (0.20, 1.52)	0.59 (0.28, 1.17)	0.60 (0.27, 1.40)	
**eGFR (CDK-EPI) (ml/min/1.73m^2)**				0.538
Median (Q1, Q3)	104.00 (87.50, 119.25)	106.00 (85.50, 121.75)	105.50 (86.75, 121.00)	
**Red cells urine (400x)**				0.580
Median (Q1, Q3)	1.00 (0.00, 5.00)	0.00 (0.00, 5.00)	1.00 (0.00, 5.00)	
**C3 (g/L)**				0.935
Median (Q1, Q3)	87.00 (67.00, 100.00)	84.50 (69.00, 104.25)	86.00 (69.00, 102.50)	
**C4 (g/L)**				0.942
Median (Q1, Q3)	16.00 (11.00, 21.25)	17.00 (11.00, 20.00)	17.00 (11.00, 21.00)	
**Ongoing therapy at 1 year**				
**Steroid dose (mg/day)**				0.653
Median (Q1, Q3)	7.50 (5.00, 10.00)	7.50 (5.00, 12.50)	7.50 (5.00, 12.50)	
**Immunosuppressive regimen (n, %)**				**< 0.001**
No immunosuppressive regimen (never started)	22 (26.1%)	8 (8.8%)	30 (18.4%)	**< 0.001**
No immunosuppressive regimen (stopped)	16 (19.0%)	4 (4.4%)	20 (10.3%)	**0.008**
CYC	8 (9.6%)	5 (5.4%)	13 (7.4%)	0.759
AZA	8 (9.6%)	13 (14.3%)	21 (12.0%)	0.361
MMF	21 (25.0%)	52 (57.1%)	73 (41.7%)	**< 0.001**
CyA	6 (7.1%)	6 (6.6%)	12 (6.9%)	1.000
Others	3 (3.6%)	3 (3.4%)	6 (3.3%)	1.000

^ normal or near-normal (within 10% of normal eGFR if previously abnormal) kidney function and proteinuria <0.5 g per day, no active extrarenal manifestations

° normal or near-normal (within 10% of normal eGFR if previously abnormal) kidney function and <50% decrease of baseline proteinuria, or proteinuria <3.5 g per day if nephrotic, no active extrarenal manifestations

Continuous variables are reported as median (IQR), categorical variables as number (%). eGFR, estimated glomerular filtration rate; CDK-EPI, Chronic Kidney Disease Epidemiology Collaboration formula; ISN, International Society of Nephrology; RPS, Renal Pathology Society; MMF, Mycophenolate Mofetil; CYC, Cyclophosphamide; AZA, Azathioprine; CyA, Cyclosporine; MLN, Membranous Lupus Nephritis.

Supplementary Table 2 – Baseline characteristics of a cohort of MLN patients at the time of kidney biopsy – patients which had withdrawn the immunosuppressive regimen within 1 year (n=22) and those which have never started the immunosuppressive regimen (n=28). Data from the overall cohort, the pure (ISN/RPS[1] class V) and the mixed (ISN/RPS[1] classes V+III and V+IV) subgroups. **(a)** Demographic, kidney features; **(b)** Extrarenal involvement, induction therapy; **(c)** Outcomes.

(a)

	Pure MLN (n=38)	Mixed MLN (n=12)	Total (N=50)	p value
**Race (n, %)**				1.000
Caucasian	34 (89.5%)	11 (91.7%)	45 (90.0%)	
African	1 (2.6%)	0 (0.0%)	1 (2.0%)	
**Sex (n, %)**				1.000
Female	35 (92.1%)	12 (100.0%)	47 (94.0%)	
**Time from SLE to kidney diagnosis (yr)**				**0.035**
Median (Q1, Q3)	0.00 (0.00, 1.90)	2.04 (0.03, 8.38)	0.02 (0.00, 2.92)	
**Age at SLE diagnosis (yr)**				0.803
Median (Q1, Q3)	24.66 (16.88, 33.93)	24.93 (16.77, 38.48)	24.66 (16.72, 35.26)	
**Activity index ISN/RPS***				**<0.001**
Median (Q1, Q3)	0.50 (0.00, 2.00)	5.00 (4.00, 7.75)	1.00 (0.00, 3.75)	
**Chronicity index ISN/RPS***				**0.026**
Median (Q1, Q3)	1.00 (0.00, 2.25)	2.50 (1.25, 4.50)	1.00 (0.00, 3.00)	
**Kidney disease (n, %)**				0.474
Urinary abnormalities	20 (52.6%)	4 (33.3%)	24 (48.0%)	
Nephrotic syndrome	17 (44.7%)	8 (66.7%)	25 (50.0%)	
Nephritic syndrome	1 (2.6%)	0 (0.0%)	1 (2.0%)	
**eGFR (CDK-EPI) (ml/min/1.73m^2)**				0.776
Median (Q1, Q3)	111.00 (77.75, 125.00)	109.50 (94.00, 115.75)	110.00 (83.25, 121.75)	
**Proteinuria (g/day)**				0.275
Median (Q1, Q3)	2.92 (1.55, 4.97)	4.66 (2.96, 5.12)	3.41 (1.75, 5.00)	
**Red cells urine (400x)**				0.740
Median (Q1, Q3)	5.00 (1.00, 10.00)	5.00 (2.00, 10.50)	5.00 (1.50, 10.00)	
**Hypertension (n, %)**	17 (44.7%)	6 (50.0%)	23 (46.0%)	1.000
**Albumin (g/dL)**				0.678
Median (Q1, Q3)	3.15 (2.40, 3.60)	3.00 (2.55, 3.35)	3.10 (2.45, 3.60)	
**C3 (g/L)**				0.789
Median (Q1, Q3)	71.00 (60.00, 90.00)	74.50 (53.00, 84.00)	71.00 (58.50, 90.00)	
**C4 (g/L)**				0.124
Median (Q1, Q3)	14.00 (12.00, 22.40)	10.50 (8.88, 16.00)	14.00 (10.00, 22.00)	
**Anti-dsDNA (n,%)**	26 (70.3%)	11 (91.7%)	37 (75.5%)	0.434
**Antiphospholipid (n, %)**	17 (47.2%)	6 (40.0%)	23 (45.1%)	0.761

*ISN/RPS [1]

Continuous variables are reported as median (IQR), categorical variables as number (%). eGFR, estimated glomerular filtration rate; CDK-EPI, Chronic Kidney Disease Epidemiology Collaboration formula; ISN, International Society of Nephrology; RPS, Renal Pathology Society; MLN, Membranous Lupus Nephritis.

(b)

	Pure MLN (n=38)	Mixed MLN (n=12)	Total (N=50)	p value
**Organ involvement (n, %)**				
Lymph nodes	3 (7.9%)	1 (9.1%)	4 (8.2%)	0.97
Skin	23 (60.5%)	6 (50.0%)	29 (58.0%)	0.51
Joints	22 (57.9%)	10 (83.3%)	32 (64.0%)	0.11
Serous membranes	6 (15.8%)	2 (16.7%)	8 (16.0%)	0.94
Central Nervous System	2 (5.3%)	0 (0.0%)	2 (4.1%)	
Fever	18 (47.4%)	7 (63.6%)	25 (51.0%)	0.30
**No steroids at 1 year (n, %)**	11(31.4%)	0 (0.0%)	11 (23.4%)	**0.03**
**Steroids dose mg/die**	7.50 (0.00, 10.62)	12.50 (10.00, 16.25)	10.00 (5.00, 12.50)	**0.021**

Continuous variables are reported as median (IQR), categorical variables as number; MLN, Membranous Lupus Nephritis.

(c)

	Pure MLN (n=38)	Mixed MLN (n=12)	Total (N=50)	p value
**Renal response at 1 year**				0.15
Complete renal response^	18 (51.4%)	5 (45.5%)	23 (50.0%)	
Partial renal response°	10 (28.6%)	6 (54.5%)	16 (34.8%)	
No renal response	7 (20.0%)	0 (0.0%)	7 (15.2%)	
**CKD at the last observation**	9 (23.7%)	0 (0.0%)	9 (18.8%)	**0.09**
**KF/Death at the last observation**	10 (26.3%)	0 (0.0%)	10 (20.0%)	**0.047**
**Flares**	11 (40.7%)	6 (54.5%)	17 (44.7%)	0.44

Continuous variables are reported as median (IQR), categorical variables as number (%). CDK, Chronic Kidney Disease; KF, Kidney Failure; MLN, Membranous Lupus Nephritis.

Supplementary Table 3 – Sustained complete renal response in a cohort of MLN patients.

Data from the overall cohort, the pure (ISN/RPS[1] class V) and the mixed (ISN/RPS[1] classes V+III and V+IV) MLN subgroups.

	Pure MLN (n=96)	Mixed MLN (n=99)	Total (N=195)	p value
**Sustained complete response***	71 (74.0%)	72 (72.7%)	143 (73.3%)	0.684
**Time to 1° sustained complete renal response (yr)**				0.513
Median (Q1, Q3)	1.45 (0.86, 5.30)	1.51 (0.95, 2.92)	1.50 (0.90, 3.56)	
**Steroid dose (mg/day)**				0.887
Median (Q1, Q3)	6.25 (5.00, 10.00)	5.00 (5.00, 10.00)	6.25 (5.00, 10.00)	
**Immunosuppressive regimen (n, %)**				**0.010**
(no immunosuppressive regimen)	29 (43.3%)	13 (18.6%)	42 (30.7%)	**0.002**
CYC	3 (4.5%)	1 (1.4%)	4 (2.9%)	0.309
AZA	8 (11.9%)	9 (12.9%)	17 (12.4%)	1.000
MMF	16 (23.9%)	32 (45.7%)	48 (35.0%)	**0.011**
CyA	7 (10.4%)	12 (17.1%)	19 (13.9%)	0.325
Others	4 (6.0%)	3 (4.3%)	7 (5.1%)	0.714
**Duration of sustained complete response**				0.536
Median (Q1, Q3)	5.55 (2.43, 11.29)	4.73 (2.44, 7.90)	5.07 (2.43, 9.46)	
**Flare (n, %)**	35 (49.3%)	35 (51.5%)	70 (50.4%)	0.342
〉 Nephritic flare	4 (5.6%)	0 (0.0%)	4 (2.9%)	
〉 Proteinuric flare	20 (28.2%)	24 (35.3%)	44 (31.7%)	
〉 Mixed flare	1 (1.4%)	2 (2.9%)	3 (2.2%)	
〉 Extrarenal flare	10 (14.1%)	9 (13.2%)	19 (13.7%)	

*the achievement of complete renal response for at least 1 year

Continuous variables are reported as median (IQR), categorical variables as number (%). ISN, International Society of Nephrology; RPS, Renal Pathology Society; MMF, Mycophenolate Mofetil; CYC, Cyclophosphamide; AZA, Azathioprine; CyA, Cyclosporine; MLN, Membranous Lupus Nephritis.

Supplementary Table 4 – Baseline characteristics of a cohort of MLN patients at the time of kidney biopsy – patients with a kidney biopsy performed between 1968-1999. Data from the overall cohort, the pure (ISN/RPS[1] class V) and the mixed (ISN/RPS[1] classes V+III and V+IV) subgroups. **(a)** Demographic, kidney features; **(b)** Extrarenal involvement, induction therapy.

(a)

	Kidney biopsy timing between 1968 and 1999			
	Pure MLN (n=43)	Mixed MLN (n=18)	Total (N=61)	p value
**Race (n, %)**				1.000
Caucasian	42 (97.7%)	18 (100.0%)	60 (98.4%)	
African	1 (2.3%)	0 (0.0%)	1 (1.6%)	
**Sex (n, %)**				1.000
Female	39 (90.7%)	17 (94.4%)	56 (91.8%)	
**Time from SLE to kidney diagnosis (yr)**				0.065
Median (Q1, Q3)	0.00 (0.00, 2.50)	1.52 (0.28, 3.77)	0.41 (0.00, 3.00)	
**Age at SLE diagnosis (yr)**				0.327
Median (Q1, Q3)	25.47 (18.29, 32.01)	21.31 (15.73, 29.74)	24.28 (17.29, 30.25)	
**Activity index ISN/RPS***				**<0.001**
Median (Q1, Q3)	0.00 (0.00, 1.50)	7.00 (4.00, 8.00)	1.00 (0.00, 4.00)	
**Chronicity index ISN/RPS***				**0.058**
Median (Q1, Q3)	1.00 (0.00, 2.00)	3.00 (0.50, 4.00)	1.00 (0.00, 3.00)	
**Kidney disease (n, %)**				0.268
Urinary abnormalities	17 (41.5%)	5 (27.8%)	22 (37.3%)	
Nephrotic syndrome	24 (58.5%)	12 (66.7%)	36 (61.0%)	
Nephritic syndrome	0 (0.0%)	1 (5.6%)	1 (1.7%)	
**eGFR (CDK-EPI) (ml/min/1.73m^2)**				0.699
Median (Q1, Q3)	106.00 (80.50, 125.00)	110.00 (95.25, 124.25)	107.00 (87.50, 125.00)	
**Proteinuria (g/day)**				**0.022**
Median (Q1, Q3)	3.40 (1.60, 5.30)	4.77 (3.25, 6.15)	3.80 (2.35, 5.47)	
**Red cells urine (400x)**				0.132
Median (Q1, Q3)	5.00 (1.00, 10.00)	13.00 (3.00, 25.00)	7.00 (1.75, 15.00)	
**Hypertension (n, %)**	22 (53.7%)	6 (35.3%)	28 (48.3%)	0.255
**Albumin (g/dL)**				0.663
Median (Q1, Q3)	3.00 (2.45, 3.60)	2.97 (2.66, 3.24)	3.00 (2.52, 3.60)	
**C3 (g/L)**				**0.005**
Median (Q1, Q3)	71.00 (60.00, 91.00)	57.00 (49.25, 69.00)	69.00 (52.88, 86.00)	
**C4 (g/L)**				**0.013**
Median (Q1, Q3)	15.00 (11.38, 24.25)	10.00 (8.00, 14.75)	14.00 (10.00, 21.75)	
**Anti-dsDNA (n,%)**	30 (73.2%)	16 (88.9%)	46 (78.0%)	0.307
**Antiphospholipid (n, %)**	17 (47.2%)	6 (40.0%)	23 (45.1%)	0.761

*ISN/RPS [1]

Continuous variables are reported as median (IQR), categorical variables as number (%). eGFR, estimated glomerular filtration rate; CDK-EPI, Chronic Kidney Disease Epidemiology Collaboration formula; ISN, International Society of Nephrology; RPS, Renal Pathology Society; MLN, Membranous Lupus Nephritis.

(b)

	Kidney biopsy timing between 1968 and 1999		Total (N=61)	p value
	Pure MLN (n=43)	Mixed MLN (n=18)		
**Organ involvement (n, %)**				
Lymph nodes	5 (11.6%)	2 (11.1%)	7 (11.5%)	1.000
Skin	23 (53.5%)	10 (55.6%)	33 (54.1%)	1.000
Joints	24 (55.8%)	13 (72.2%)	37 (60.7%)	0.265
Serous membranes	8 (18.6%)	5 (27.8%)	13 (21.3%)	0.499
Central Nervous System	3 (7.0%)	1 (5.6%)	4 (6.6%)	1.000
Fever	19 (44.2%)	9 (50.0%)	28 (45.9%)	0.781
**No treatment (n, %)**	5 (11.6%)	0 (0%)	5 (8.2%)	0.318
**Steroids (n, %)**				**0.020**
(no steroids)	8 (18.6%)	1 (5.6%)	9 (14.8%)	0.259
Steroids ev + oral	26 (60.5%)	16 (88.9%)	42 (68.9%)	**0.035**
Steroids oral only	9 (20.9%)	0 (0.0%)	9 (14.8%)	**0.047**
Steroids oral (ev not known)	0 (0.0%)	1 (5.6%)	1 (1.6%)	0.295
**Immunosuppressive regimen – induction (n, %)**				
(no induction regimen)	15 (34.9%)	6 (33.3%)	21 (34.4%)	1.000
CYC	21 (48.8%)	9 (50.1%)	30 (49.1%)	1.000
AZA	3 (7.0%)	2 (11.1%)	5 (8.2%)	0.626
MMF	1 (2.3%)	0 (0.0%)	1 (1.6%)	1.000
CyA	1 (2.3%)	1 (5.6%)	2 (3.3%)	0.506
Others	2 (4.7%)	0 (0.0%)	2 (3.3%)	1.000
**Hydroxychloroquine (n, %)**	4 (10.3%)	1 (6.7%)	5 (9.3%)	1.000

*ISN/RPS [1]

Continuous variables are reported as median (IQR), categorical variables as number (%). ISN, International Society of Nephrology; RPS, Renal Pathology Society; MLN, Membranous Lupus Nephritis.

Supplementary Table 5 – Baseline characteristics of a cohort of MLN patients at the time of kidney biopsy – patients with a kidney biopsy performed between 2000-2023. Data from the overall cohort, the pure (ISN/RPS[1] class V) and the mixed (ISN/RPS[1] classes V+III and V+IV) subgroups. **(a)** Demographic, kidney features; **(b)** Extrarenal involvement, induction therapy.

(a)

	Kidney biopsy timing between 2000 and 2023			
	**Pure MLN (n=53)**	**Mixed MLN (n=81)**	**Total (N=134)**	**p value**
**Race (n, %)**				0.748
**Caucasian**	43 (81.1%)	60 (74.1%)	103 (76.9%)	
**American Indian**	4 (7.5%)	9 (11.1%)	13 (9.7%)	
**Asian**	4 (7.5%)	6 (7.4%)	10 (7.5%)	
**African**	2 (3.8%)	6 (7.4%)	8 (6.0%)	
**Sex (n, %)**				1.000
Female	42 (79.2%)	65 (80.2%)	107 (79.9%)	
**Time from SLE to kidney diagnosis (yr)**				0.554
Median (Q1, Q3)	0.80 (0.00, 5.92)	1.37 (0.08, 7.13)	1.04 (0.00, 7.01)	
**Age at SLE diagnosis (yr)**				0.895
Median (Q1, Q3)	25.55 (20.39, 37.95)	25.59 (20.54, 38.32)	25.57 (20.39, 38.23)	
**Activity index ISN/RPS***				**<0.001**
Median (Q1, Q3)	0.00 (0.00, 1.50)	6.00 (4.00, 8.00)	4.00 (1.00, 7.00)	
**Chronicity index ISN/RPS***				**0.003**
Median (Q1, Q3)	0.00 (0.00, 1.00)	2.00 (1.00, 3.50)	1.00 (0.00, 2.75)	
**Kidney disease (n, %)**				**0.056**
Urinary abnormalities	28 (52.8%)	36 (45.0%)	64 (48.1%)	0.41
Nephrotic syndrome	24 (45.3%)	31 (38.8%)	55 (41.4%)	0.49
Nephritic syndrome	1 (1.9%)	13 (16.3%)	14 (10.5%)	**0.08**
**eGFR (CDK-EPI) (ml/min/1.73m²)**				**0.045**
Median (Q1, Q3)	114.00 (95.00, 124.50)	104.00 (86.00, 118.00)	105.50 (91.00, 119.75)	
**Proteinuria (g/day)**				0.407
Median (Q1, Q3)	2.85 (1.89, 5.23)	3.70 (2.27, 5.50)	3.28 (1.90, 5.46)	
**Red cells urine (400x)**				**0.016**
Median (Q1, Q3)	4.00 (1.00, 17.00)	15.00 (5.00, 30.00)	10.00 (2.50, 29.00)	
**Hypertension (n, %)**	8 (16.0%)	33 (41.8%)	41 (31.8%)	**0.004**
**Albumin (g/dL)**				0.098
Median (Q1, Q3)	2.75 (2.23, 3.27)	2.90 (2.48, 3.54)	2.90 (2.40, 3.40)	
**C3 (g/L)**				0.355
Median (Q1, Q3)	74.00 (54.50, 93.75)	69.00 (49.50, 87.00)	70.00 (51.00, 90.00)	
**C4 (g/L)**				0.1
Median (Q1, Q3)	12.50 (7.25, 19.75)	10.00 (6.00, 15.00)	11.00 (6.00, 17.50)	
**Anti-dsDNA (n,%)**	34 (68.0%)	68 (86.1%)	102 (79.0%)	**0.016**
**Antiphospholipid (n, %)**	11 (23.4%)	19 (25.0%)	30 (24.4%)	0.84

*ISN/RPS [1]

Continuous variables are reported as median (IQR), categorical variables as number (%). eGFR, estimated glomerular filtration rate; CDK-EPI, Chronic Kidney Disease Epidemiology Collaboration formula; ISN, International Society of Nephrology; RPS, Renal Pathology Society; MLN, Membranous Lupus Nephritis.

(b)

	Kidney biopsy timing between 2000 and 2023			
	**Pure MLN (n=53)**	**Mixed MLN (n=81)**	**Total (N=134)**	**p value**
**Organ involvement (n, %)**				
Lymph nodes	5 (10.0%)	11 (13.9%)	16 (12.4%)	0.52
Skin	30 (58.8%)	43 (53.1%)	73 (55.3%)	0.53
Joints	34 (66.6%)	64 (80.0%)	98 (75.0%)	0.10
Serous membranes	18 (35.3%)	17 (21.0%)	35 (26.5%)	0.086
Central Nervous System	2 (3.9%)	5 (6.3%)	7 (5.4%)	0.56
Fever	24 (47.1%)	30 (38.0%)	54 (41.5%)	0.31
**No treatment (n, %)**	1 (1.9%)	0 (0%)	1 (0.7%)	0.830
**Steroids (n, %)**				0.105
(no steroids)	1 (1.9%)	3 (3.7%)	4 (3.0%)	
Steroids ev + oral	37 (69.8%)	66 (81.5%)	103 (76.9%)	
Steroids oral only	5 (9.4%)	1 (1.2%)	6 (4.5%)	
Steroids oral (ev not known)	10 (18.9%)	11 (13.6%)	21 (15.7%)	
**Immunosuppressive regimen – induction (n, %)**				
(no induction regimen)	7 (13.2%)	1 (1.2%)	8 (6.0%)	**0.0067**
CYC	17 (32.1%)	34 (42.0%)	51 (38.1%)	0.275
AZA	3 (5.7%)	1 (1.2%)	4 (3.0%)	0.300
MMF	19 (35.8%)	38 (46.9%)	57 (42.5%)	0.212
CyA	3 (5.7%)	2 (2.5%)	5 (3.7%)	0.386
Others	4 (7.6%)	4 (5.0%)	8 (6.0%)	1.000
**Hydroxychloroquine (n, %)**	22 (44.0%)	45 (57.0%)	67 (51.9%)	0.205

*ISN/RPS [1]

Continuous variables are reported as median (IQR), categorical variables as number (%). eGFR, estimated glomerular filtration rate; CDK-EPI, Chronic Kidney Disease Epidemiology Collaboration formula; ISN, International Society of Nephrology; RPS, Renal Pathology Society; MLN, Membranous Lupus Nephritis.

Supplementary Figures

Supplementary Figure 1 – Crude patient's relapse free-survival (a) and proteinuric flare free-survival (b) (Kaplan-Meier estimator) in patients with pure MLN (blue line) and in those with mixed MLN (red line). P value refers to Log-rank test, which is based on the time to the latest available follow-up, though the plot refers to the first 20 years. Relapses were defined as a nephritic flare or proteinuric flare or extrarenal flare in patients which had previously reached CR (which has a duration of at least 1 year).

MLN, Membranous Lupus Nephritis.

Supplementary Figure 2 – Crude patient's CKD free-survival (a) and CKD/Death free-survival (b) (Kaplan-Meier estimator) in patients with pure MLN (blue line) and in those with mixed MLN (red line). P value refers to Log-rank test, which is based on the time to the latest available follow-up, though the plot refers to the first 30 years.

MLN, Membranous Lupus Nephritis; CKD, Chronic Kidney Disease.
